# The Chemical and Biological Profiles of Leaves from Commercial Blueberry Varieties

**DOI:** 10.3390/plants9091193

**Published:** 2020-09-12

**Authors:** Bianca-Eugenia Ștefănescu, Lavinia Florina Călinoiu, Floricuța Ranga, Florinela Fetea, Andrei Mocan, Dan Cristian Vodnar, Gianina Crișan

**Affiliations:** 1 Department of Pharmaceutical Botany, “Iuliu Hațieganu” University of Medicine and Pharmacy, Ghe. Marinescu Street 23, 400337 Cluj-Napoca, Romania; stefanescu.bianca@umfcluj.ro (B.-E.Ș.); mocan.andrei@umfcluj.ro (A.M.); gcrisan@umfcluj.ro (G.C.); 2 Institute of Life Sciences, University of Agricultural Sciences and Veterinary Medicine Cluj-Napoca, Calea Mănăștur 3–5, 400372 Cluj-Napoca, Romania; 3 Faculty of Food Science and Technology, University of Agricultural Sciences and Veterinary Medicine Cluj-Napoca, Calea Mănăștur 3–5, 400372 Cluj-Napoca, Romania; floricuta.ranga@usamvcluj.ro (F.R.); florinela.fetea@usamvcluj.ro (F.F.); 4 Laboratory of Chromatography, Institute of Advanced Horticulture Research of Transylvania, University of Agricultural Sciences and Veterinary Medicine, 400372 Cluj-Napoca, Romania

**Keywords:** blueberry, leaves, varieties, polyphenolic compounds, biological profile, antioxidant capacity, antimicrobial activity, antimutagenicity

## Abstract

Blueberries have seen an ascending production line boosted by World Health Organization (WHO) approvals for their contributions to a healthy diet and the evidence that they act against different diseases. This increase resulted in significant amounts of discarded leaves, which could be a valuable source of bioactive compounds. In the present study, ultrasound-assisted extraction technology was used to determine and compare the chemical and biological profiles of leaves from six commercial blueberry (*Vaccinium corymbosum* L.) varieties. Feruloylquinic acid was the major compound identified, ranging from 19.23 ± 0.18 mg/g (at the lowest level, registered in the *Spartan* variety) to 49.62 ± 0.41 mg/g (at the highest level, registered in the *Nelson* variety). Rutin was the second major compound identified, for which *Toro*, *Nelson*, and *Elliot* leaves registered the highest values, with 35.77 ± 0.19 mg/g, 32.50 ± 0.20 mg/g, and 31.53 ± 0.1 mg/g, respectively. Even though analogous polyphenols were detected in the six cultivars, their concentrations and amounts were different. The leaf extracts of the cultivars *Toro*, *Elliot*, and *Nelson* appear to be good sources of antioxidants, registering high percentage inhibitions of DPPH radicals, of 70.41%, 68.42%, and 58.69%, respectively. The blueberry leaf extracts had a strong antibacterial activity and a low antifungal capacity, and a low-to-moderate antimutagenic capacity towards *Salmonella typhimurium* TA98 and TA100 strains, with *Toro* leaf being the best candidate. All of these biological activities indicate health-related benefits, recommending them as suitable candidates for medical and pharmaceutical applications. The present paper adds significant knowledge to the field of blueberry leaves via chemical and biological profiles, supporting the ultrasound-assisted extraction technique as a useful and green method to provide alternative sources of bioactive compounds.

## 1. Introduction

Current evidence has underlined the fact that the overproduction of reactive oxygen species (ROS) and free radicals may be responsible for several pathological reactions that could contribute to the occurrence of specific diseases such as cancer, atherosclerosis, diabetes, and rheumatoid arthritis [1,2,3]. Moreover, more bacteria have become resistant to well-established antibiotics, underlying the emergent need for novel/natural anti-microbial molecules [4,5,6,7,8]. The plant kingdom is a rich complex of bioactive compounds [9,10,11,12,13]. In this context, plant-based antioxidant compounds have received increased attention due to their ability to protect the human body against free radicals without—or with fewer—side effects [14,15]. Several species of the Vaccinium genus represent extensively used tools in traditional medicines for the treatment of diabetic symptoms [16,17]. The *Vaccinium corymbosum* (*V. corymbosum*) plant, also known as blueberry, has been reported as a complex of antioxidant and antimicrobial molecules—more precisely, polyphenol compounds—with a wide range of anthocyanins [18,19,20,21]. The Blueberry, Vaccinium spp., is a perennial shrub which is well-known worldwide, whereas the northern highbush blueberry (*V. corymbosum* L.) is the most popular [22,23,24].

Numerous reports have detailed the *V. corymbosum* L. (blueberry) fruit’s benefits for human health [22,25]. It has been demonstrated to protect against cancer [26,27] stroke [28] and urinary tract disease [29]. Furthermore, another study has shown that a blueberry-abundant diet can improve short-term memory loss and re-establish the loss of balance and coordination in aging rats [30]. A more recent paper has concluded that blueberry may have a positive effect in preventing cancer development and heart diseases by decreasing the cancer cell proliferation, considering existing studies, both in vitro and in vivo [31]. Such evidence has placed blueberries (*V. corymbosum*) in the top rank of the most popular berries [32] and, considering the worldwide recommendations for fruits and vegetable intakes [33], boosted their production and consumption. Therefore, significant quantities of *Vaccinium* leaves are also generated.

Blueberries (*V. corymbosum* L.) are a rich source of flavonoids and phenolic acids, which are known for their biological activities, e.g., antimicrobial activities [34,35,36] and high radical scavenging capacity [37,38,39]. The formation and accumulation of bioactive compounds in the fruits are influenced by several factors, such as genotype, environmental conditions, ripeness, and storage [37,40,41,42]. Moreover, phenolic compounds are particularly sensitive to environmental stress, and their content changes rapidly under the influence of stress [43]. Furthermore, recent studies have shown that the levels of anthocyanins, chlorogenic acid, and quercetin were influenced by the blueberry cultivar, as well [34,44]. The berries (and leaves) of *Vaccinium* L. (Ericaceae family) represent a significant source of food and pharmaceutical ingredients [45,46], whereas numerous products made from *Vaccinium* leaf and fruit extracts, commercialized as dietary supplements in the world market, have also been reported [47].

While the *Vaccinium corymbosum* (*V. corymbosum* L.) fruits (blueberries) are well characterized, the information available about the chemical and biological profiles of their leaves is limited [35,48,49]. There is a lack of knowledge on the antimutagenic, antibacterial, and antioxidant activities of hydroalcoholic leaf extracts from commercial *V. corymbosum* L. varieties. These may provide an alternative and cheap bioactive source that can be used as references in analytical research, and as a top source of health-related compounds for medical and pharmaceutical applications [50].

Blueberry (*V. corymbosum* L.) leaves are the aerial parts of the plant, which have been characterized by their medicinal uses for centuries [51]. The leaves have varying phenolic compositions during different seasons; this fact is illustrated in the changing of their color from green to red in the autumn. The existing literature has demonstrated that the leaf extract of *Vaccinium angustifolium* (*V. angustifolium*) is rich in chlorogenic acid, several flavonols, and glycosides, as well as catechin and epicatechin [52,53]. Therefore, blueberry leaves can be described as an abundant and readily available source of 5-caffeoylquinic acid [51]. A recent study reported that leaf-derived proanthocyanidin from a particular blueberry cultivar (*V. virgatum* Aiton) proved to have strong suppressive effects towards Hepatitis C virus subgenome expression in a replicon cell system [54].

Considering the above, in the present study, the phenolics, anthocyanins, and flavonoids contents were extracted via the ultrasound-assisted extraction technique, and were determined for the leaves of six commercial blueberry varieties, all of which had the same geographical origin (North-West of Romania, same producer) and were kept under several controlled environmental factors (soil, irrigation). The comparisons among the cultivars aimed to evaluate the differences between the polyphenolic compositions of the leaves, and to analyze their biological profile—such as their antioxidant, antimicrobial, and antimutagenic activities—thus adding valuable novelty to the present paper. Such research should be of use in the investigation of biological and chemical profiles to enhance the use of leaves (from the most commercialized blueberry varieties) in medical and pharmaceutical applications.

## 2. Materials and Methods

### 2.1. Plant Materials

Six Romanian blueberry cultivar varieties with the same provenience—namely Orăștie city, a northwestern location in Romania (45°51′ N, 23°12′ E)—and similar cultivations (controlled drip irrigation system, acid brown soil) were used in the present study: *Elliot*, *Toro*, *Duke*, *Bluecrop*, *Spartan*, *Nelson*. The blueberry leaves of these varieties were collected in September 2017, followed by drying (7–10 days, room temperature) and being ground to obtain a fine powder, and dark storage until the analyses.

### 2.2. Chemicals

Reference compounds—namely the catechin, chlorogenic acid, quercetin, cyanidin chloride, and gallic acid—were used for the HPLC-DAD-MS (Sigma-Aldrich, Steinheim, Germany). The chemical reagents for the analytical methods involved (Folin-Ciocalteu, total flavonoids content, HPLC, DPPH antioxidant activity) were also obtained from the same supplier. The specific culture media for the antimicrobial testing were bought from BioMerieux (Craponne, France) and Sigma-Aldrich (Steinheim, Germany).

### 2.3. Biomolecules Extraction Assisted by Ultrasounds

The ultrasound-assisted extraction (Elmasonic E15H, Elma, Singen, Germany) of the leaf powder (0.25 g) used 7 mL 40% *v*/*v* ethanol/water for 30 min at 20 °C, at an ultrasonic frequency of 37 kHz, followed by their centrifugation (5000 rpm, 10 min at 24 °C), filtration, the collection of the supernatant, and storage (−18 °C) prior chemical and biological analysis.

### 2.4. Evaluation of Phenolic Profile and Content

#### 2.4.1. HPLC-DAD-ESI-MS Chemical Profile

All the leaf extracts belonging to the selected six blueberry cultivars were subjected to HPLC-DAD-ESI-MS analysis to identify and quantify their phenolic compounds. A system composed of an Agilent 1200 HPLC with a DAD detector and an MS-detector single-quadrupole Agilent 6110 was used. The following XDB C18 Eclipse column (4.6 × 150 mm, particle size 5 µm) (Agilent Technologies, USA) was selected for the phenolic separation at 25 °C. Two gradients composed of 0.1% acetic acid in distilled water (*v*/*v*) (solvent A) and 0.1% acetic acid in acetonitrile (*v*/*v*) (solvent B) were used at a flow rate of 0.5 mL/min, following the elution program detailed by Dulf et al. [55]. A scanning range of 100–1200 m/z in the ESI (+) mode was applied for the MS fragmentation, as well as setting the capillary voltage at 3000 V, 350 °C, and the nitrogen flow at 8 L/min. DAD was used to monitored the eluent, while the spectra of absorbance (200–600 nm) were registered during each run. Agilent ChemStation Software (Rev B.04.02 SP1, Palo Alto, CA, USA) was used to analyze the data. A comparison between the retention times, the visible UV, and the mass spectra of the peaks with 4 reference standards was performed for the accurate and correct identification of each phenolic compound, as follows: the anthocyanins were determined via the cyanidin calibration curve (concentration ranges 10–100 µg/mL), which were reported as cyaniding equivalents (mg cyanidin/g plant material) (r^2^ = 0.9951); the chlorogenic acid calibration curve (10–50 µg/mL concentrations) was used to quantify the hydroxycinnamic acid compounds (mg chlorogenic acid/g plant material) (r^2^ = 0.9937); the compounds from the flavonol group were reported as quercetin equivalents (mg quercetin/g plant material) (r^2^ = 0.9951) using the quercetin calibration curve (10–200 µg/mL concentrations); the compounds of the flavanol subclass were reported as equivalents of catechin (mg catechin/g plant material) (r^2^ = 0.9985) using the catechin standard for the calibration curve (10–200 µg/mL concentrations).

#### 2.4.2. The Total Content of Phenolics

The total phenolic content (TPC) was determined based on the well-known spectrophotometric Folin–Ciocalteu method [55,56]. As such, 125 µL Folin–Ciocalteu reagent (0.2 N) was added to 25 µL of leaf extract and mixed with 100 µL sodium carbonate solution (Na_2_CO_3_, 7.5% *w*/*v*). The obtained mixture was placed in the dark for 2 h at room temperature (25 °C), followed by the absorbance measurement at 760 nm, with ethanol as a blank. To express the TPC of the leaf extracts, a gallic acid calibration curve (0.01–1 mg/mL) was performed, and the results were reported as gallic acid equivalents (GAE) (mg GAE/100 g plant material).

#### 2.4.3. The Total Content of Flavonoids

The spectrophotometric method was used to calculate the total flavonoid content (TFC), following a previously described protocol [57], considering the aluminum chloride colorimetric assay and using quercetin as a reference standard. An exact volume of 1 mL leaf extract was added to 0.3 mL NaNO_2_ (5%) solution, and after 5 min, the addition of 0.3 mL AlCl_3_ (10%) was performed. In the end, 2 mL NaOH (1M) and water—to a total volume of 10 mL—were added, followed by an immediate absorbance measurement at 510 nm. The results were reported as quercetin equivalents (QE) (mg QE/100 g plant material).

#### 2.4.4. The Total Content of Anthocyanins

The previously described spectrophotometric method [58] was used to calculate the total anthocyanin content (TAC). The UV-VIS spectra (Jasco V-530 double beam UV-Visible Spectrophotometer, Tokyo, Japan) were registered for each hydroethanolic extract, at 530 nm. Cyanidin-3-glucoside was used to quantify the anthocyanin content with a molar absorptivity coefficient of 26,900, which was expressed as milligrams per 100 g of plant material [58].

### 2.5. DPPH Antioxidant Capacity

The spectrophotometric method detailed by Ebrahimabadi et al. [59] was performed (with a few modifications) to determine the DPPH free-radical–scavenging activity. Therefore, 1750 µL DPPH solution (0.1 mM in 40% ethanol) was mixed with 250 µL hydroethanolic leaf extract, followed by storage in dark and static conditions for 30 min. Afterward, the spectra were registered at 517 nm (Biotek) using 40% ethanol in water as a blank. The percentage inhibition (I%) was determined according to the formula: I% = [(A_B_ − A_A_)/A_B_] × 100, where A_B_ = the absorbance of the blank, and A_A_ = the absorbance of the leaf extract.

### 2.6. Antimicrobial Activity

#### 2.6.1. Microorganisms

The microorganisms used for the antimicrobial testing were received from the Food Biotechnology Laboratory (UASVM Cluj-Napoca, Romania), as follows: *Staphylococcus aureus* (ATCC 49444), *Enterococcus faecalis* (ATCC 29212), *Rhodococcus equi* (ATCC 6939), *Pseudomonas aeruginosa* (P.A) (ATCC 27853), *Klebsiella pneumonia* (K.P) (DSMZ 2026), *Escherichia coli enterotoxigen* (ETEC) (ATCC 25922).

#### 2.6.2. Microdilution Technique

The determination of the antimicrobial capacity followed a slightly adapted version of the guidelines of the Clinical Laboratory Standards Institute (CLSI) [60]. In brief, Mueller–Hinton agar was used for the bacterial culture, and then stored (4 °C) and subcultured monthly. Before the antibacterial determination, the strains were inoculated on agar plates, followed by their incubation at 37 °C for 24 h. The inoculums (density of 0.5 in the McFarland scale) were adjusted in a 0.9% NaCl sterile solution. Afterward, the bacteria were suspended in the broth (Mueller–Hinton), to reach the targeted density of 2 × 10^5^ CFU/mL, then stored at 4 °C. The minimum inhibitory concentration (MIC) determination was performed via the serial dilution technique (two-fold dilutions) using 96-well plates. Into each well of the 96 well microplate was added 100 µL Mueller-Hinton broth. Samples of 100 µL of every extract (0.1 g/mL concentration) were added and a two-fold dilution was applied. The addition of 10 µL cell suspension was added to every well. The 40% ethanol was used as a control, to report the antimicrobial effect of the sample (phenolics) without the effect provided by the solvent. The microplates’ incubation was performed for 24–48 h at 37 °C. After the incubation, the resazurin solution (20 μL) was added to each well, and then incubated (2 h, 37 °C). When the swift colour change from blue to pink appears—signalling the resazurin reduction—bacterial growth results. The minimum extract concentration that inhibited bacterial growth was defined as MIC [61], which obviated the changing of the color. The minimum bactericidal concentration (MBCs) determination were achieved via the serial subcultivation of 2 μL into 96-well plates filled with 100 μL of Mueller-Hinton broth per well, followed by their incubation at 37 °C for 48 h. The lowest extract concentration killed 99.9% of the bacteria; thus, no visible growth was described as MBC [61]. The positive control used for the bacterial growth was Streptomycin (Sigma P 7794, Santa Clara, CA, USA) (0.05–3 mg/mL), whereas the negative control was water.

#### 2.6.3. Antifungal Test

The antifungal guidelines of the Clinical Laboratory Standards Institute (CLSI) [62] were used, but slightly adapted. Three types of fungi were tested: *Candida albicans (ATCC 10231)*, *Candida zeylanoides (ATCC 20367*), and *Candida parapsilosis (ATCC 22019)*. These strains were received from the Food Biotechnology Laboratory, UASVM Cluj-Napoca, Romania. The fungi were maintained on malt agar at 4 °C and subcultured monthly. The *Candida* spp. had an initial density of around 2 × 10^6^ CFU/mL. NaCl sterile solution (0.9%) was used to adjust the inoculums to a density of 0.5 on the McFarland scale. Afterward, the studied strains were suspended in broth malt medium, to reach the targeted density of 1.5 × 10^5^ CFU/mL. The microdilution method was applied to determine the minimum inhibitory concentration, by preparing successive dilutions (two-fold dilutions) in 96-well plates. In total, 100 µL medium was placed in every well of the 96-well microplates. Samples of 100 µL of every extract, diluted in 0.85% saline (concentration of 0.1 g/mL), were placed into the first rows of the microplates, followed by two-fold serial dilutions. The 40% ethanol was used as a control, to report the antimicrobial effect of the sample (phenolics) without the effect provided by the solvent. Next, 10 µL of the bacteria suspension was placed in each well, followed by incubation at 28 °C for 72 h, with continuous shaking. The addition of resazurin (20 μL, 0.02%) to each well, and their incubation for 2 h, was performed before the minimum inhibitory concentration (MIC) determination. The MIC definition gives the lowest concentration needed to inhibit the growth of the microorganism. The minimum fungicidal concentration (MFCs) determination was performed via the serial subcultivation of 2 μL of the extracts, following their homogenization into the medium, and inoculation for 72 h into microtiter plates filled with 100 μL of broth/well and additional incubation for 72 h at 28 °C. The MFC was described as the lowest concentration with no visible growth, signifying that 99.9% of the original inoculum was killed. Fungicide fluconazole (1–3500 µg/mL) (Sigma F 8929, Santa Clara, CA, USA) was the positive control, while water was the negative one. All of the tests, in duplicate, were repeated three times.

### 2.7. Antimutagenic Activity

To test the antimutagenic capacity of the leaf extract, the plate incorporation method [63] was used; this method was described in further detail by Sarac and Sen [64]. The antimutagenic assay involved testing against *Salmonella typhimurium* TA98 and *Salmonella typhimurium* TA100. Positive controls were used for each type of strain: in the case of TA98, 4-nitro-ophenylenediamine (4-NPD, 3 mg/plate) was established, and in the case of TA100, sodium azide (NaN3, 8 mg/plate) was used. The ethanol:water (1:1, *v*/*v*) was the negative control for both strains. The leaf extract’s concentration was 5 mg/plate. In agreement with the equation presented by Ong et al. [65], there is the following antimutagenicity equation for establishing the inhibition percentage: %Inhibition = [1 − T/M] × 100, where T represents the number of revertants per plate when the mutagen and the leaf extract are present, and M represents the number of revertants per plate with no leaf extract (positive control).

In the absence of the leaf extracts, the antimutagenicity of the reference mutagens was reported as 0% inhibition, whereas in the presence of the extracts, the antimutagenicity was expressed accordingly: strong for 40% or more inhibition; moderate for 25–40% inhibition; low/none for 25% or less inhibition [66]. The assay was performed in duplicate with three subsamples each, and the data is reported as the mean ± standard deviation (SD).

### 2.8. Statistical Interpretations of Results

The data were registered as the means ± standard deviation (SD), after performing the analysis in triplicate. A one-way ANOVA via Tukey multiple comparison tests (software GraphPad Prism Version 8.0.1, Graph Pad Software Inc., San Diego, CA, USA) was applied to estimate the statistical differences between the leaf extracts of the six cultivars. Statistically significant differences between the means were reported at the 5% level.

## 3. Results and Discussion

### 3.1. Phenolic Profile of the Leaf Extracts from the Six Blueberry Varieties

In the leaves of the six Romanian blueberry cultivars were identified 19 phenolic compounds belonging to four phenolic classes: hydroxycinnamic acids, flavonols, flavanols, and anthocyanins (Table 1). The 4 hydroxycinnamic acids identified were: chlorogenic acid (5-caffeoylquinic acid), caffeic acid, feruloylquinic acid, and dicaffeoylquinic acid. Among the flavanols group, six compounds were detected: gallocatechin, catechin, epicatechin, procyanidin dimer I, procyanidin dimer II, procyanidin trimer. Furthermore, in the flavonols group, six compounds were detected, namely: quercetin-rutinoside (Rutin), quercetin-glucoside, quercetin-acetyl-rhamnoside, quercetin-arabinoside, quercetin-diglucoside, and quercetin. The anthocyanins subclass was represented by three compounds: cyanidin-glucoside, cyanidin-arabinoside, and cyanidin-acetyl-glucoside. These results are similar to the findings of Wang et al. [67], wherein 104 blueberry cultivars were studied. The HPLC-DAD-ESI-MS analysis of the methanolic extracts reported anthocyanins, flavonols, hydroxycinnamic acids, and proanthocyanidin; these findings are in accordance with the results of Riihinen et al. [36].

The best-represented subclasses in terms of the number of phenolic compounds were flavanols and flavonols, while the most abundant group in terms of the highest levels, for all six varieties, were hydroxycinnamic acids. The second most abundant class was flavanols, followed closely by flavonols. In Table 2, below, are identified and quantified all of the individual phenolic compounds in the leaf extracts of all of the six blueberry varieties.

Among all of the phenolic compounds identified, the feruloylquinic acid from the hydroxycinnamic group had the highest amount, ranging from 19.23 ± 0.18 mg/g (as the lowest level, registred for the *Spartan* variety) and 49.62 ± 0.41 mg/g (as highest level, registered for the *Nelson* variety). The *Toro* variety was placed second, with a concentration of feruloylquinic acid of 44.43 ± 0.37 mg/g. Chlorogenic acid was the minor compound identified within this group, among all the six varieties, with values of <1.5 mg/g. Interestingly, in the study of Wang et al. [67], the chlorogenic acids were the most abundant phenolic compounds in the leaves of different blueberry cultivars. Possible explanations could be found in the varieties studied (104 cultivars), extraction method, time of harvesting, or geographical area.

In the case of dicaffeoylquinic acid, again, the *Nelson* variety had the highest level (10.18 ± 0.11 mg/g), followed by the *Elliot* and *Toro* varieties. For caffeic acid, *Elliot* and *Nelson* registered similar amounts of >5 mg/g, these being he highest concentrations found.

Regarding the flavonols group, also called quercetin derivates, the major phenolic compound identified was rutin (quercetin-rutinoside), whereas the highest values were registered by *Toro*, *Nelson*, and *Elliot* leaves, with 35.77 ± 0.19 mg/g, 32.50 ± 0.20 mg/g, and 31.53 ± 0.1 mg/g, respectively. The lowest value was again reported for the *Spartan* variety, being found in half the amount of the highest level. The second major compound identified among the flavonols was quercetin–glucoside, being 10-fold lower than rutin. However, the *Toro* variety registered the highest value, at precisely 6.70 ± 0.08 mg/g. The rest of the varieties’ reported values ranged between 2–3.5 mg/g. Quercetin-arabinoside was found to be specific for *Spartan* leaves—and was missing in the other varieties studied—but in a small amount. Traces of quercetin-diglucoside were found in *Elliot* and *Toro* leaves. The highest quercetin amounts were identified in *Spartan*, *Toro*, and *Nelson* leaves, with 3.69 ± 0.04 mg/g, 3.68 ± 0.04 mg/g, and 3.56 ± 0.02 mg/g, respectively. In the study of Riihinen et al. [36], the red leaves of the *Northblue* blueberry (*V. corymbosum*) contained similar flavonols and hydroxycinnamic acids.

In the flavanols group, the procyanidin dimer II and procyanidin trimer were found in remarkable amounts for all the six varieties’ leaves. Compared to the other two phenolic groups discussed above, *Nelson* variety leaves presented the lowest amount of procyanidin dimer II (3.92 ± 0.03 mg/g), whereas the *Spartan* leaves registered the highest value (17.15 ± 0.15 mg/g), followed by *Toro* and *Elliot*. Regarding the procyanidin trimer compound, *Toro* leaves were top-leveled, with an amount of 15.99 ± 0.22 mg/g. The other varieties’ leaves contained half this amount less, except the *Nelson* variety, which registered 12.27 ± 0.15 mg/g. The epicatechin and procyanidin dimer I were identified only in the leaves of the *Elliot* variety in significant amounts, precisely 4.19 ± 0.03 mg/g and 3.69 ± 0.04 mg/g, respectively. Concerning gallocatechin and catechin compounds, the highest value was identified in *Toro* leaves, followed by *Elliot* and *Spartan* leaves. In the study of Wang et al. [67] were reported four proanthocyanidins, but in low concentrations, in *V. corymbosum* leaves (0.36–8.38 mg rutin equivalents/g dry weight). The leaves of *V. corymbosum* from Drama [68] presented the following phenolic profile: quinic and caffeic acid, proanthocyanidin B1/B2, myricetin glycosides, kaempferol rutinoside, and quercetin glycosides and quercetin aglycone, whereas rutin was among the principal flavonoids (12.09 mg/g, 4.60 mg/g). The absence of anthocyanins from the extract was reported. Another study [36] reported prodelphinidins and procyanidins in the same quantity in the red and green leaves of *V. corymbosum* (0.468–0.485 mg/g frozen sample).

The anthocyanins group, represented by the three cyanidin derivates, were present only in small amounts, precisely <1 mg/g, for only three varieties: *Toro*, *Elliot*, and *Spartan*. The order is representative of the descending amounts. These compounds are reported to be responsible for the red colour of the leaves. These results follow the findings of Wang et al. [67], who also identified three anthocyanins (cyanidin 3-O-glucoside, cyanidin 3-O-glucuronide, cyanidin 3-O- arabinoside) in the blueberry leaf methanolic extracts. The blueberry study of Riihinen et al. [36] investigated both green and red, wherein the anthocyanins were present only in red leaves—precisely, cyanidin-glycosides (62 µg/g frozen sample)—and were absent in the green ones. In the study of Li et al. [69], 13 anthocyanins were identified and quantified in six blueberry varieties, including *Duke*, *Bluecrop*, and *Spartan*, suggesting that blueberry fruits are richer in anthocyanins than their counterpart leaves.

According to Riihinen et al. [36], leaf tissue maturation is a determining factor in the blueberry’s phytochemical composition. In his study, the red leaves of *V. corymbosum* were reported to have increased levels of flavonols and hydroxycinnamic acids (quercetin, kaempferol, *p*-coumaric, caffeic and ferulic acids) compared to the green ones, concluding the possible role of solar radiation in photoprotection [70].

Knowledge of the pharmacologically active phenolics inside the plant, including leaves, and the understanding of their mechanism of action will support the development of genetically enhanced cancer-fighting plants.

### 3.2. Total Phenolic and Total Flavonoid Contents

**Figure 1:**
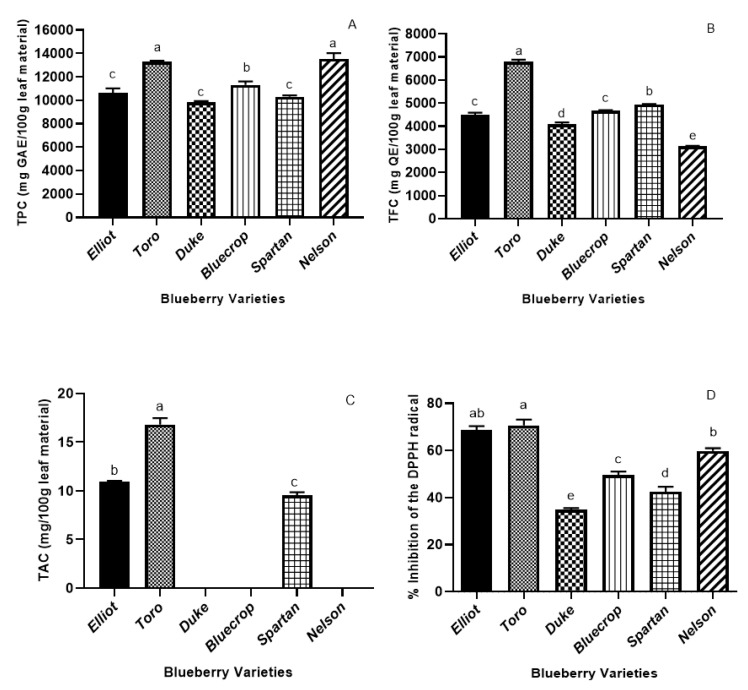
Total phenolic content (Folin–Ciocalteu method) (**A**), total flavonoid content (**B**), total anthocyanin content (**C**), and DPPH antioxidant activity (**D**) of the leaf extracts from the six commercial blueberry varieties. The total phenolic content of the extract is expressed as gallic acid equivalents (GAE) in mg/100 g leaf material. The total flavonoid content is expressed as quercetin equivalents (QE) in mg/100 g leaf material. Values (means ± SD, *n* = 3) followed by different letters (a–e) indicate significant differences (*p* < 0.05) between the six varieties (One-way ANOVA-Tukey multiple range tests).

The TPCs of the six blueberry varieties’ leaves are illustrated in Figure 1A. The *Nelson* and *Toro* cultivars registered the highest TPC, precisely 13,555 mg GAE/100 g leaf material and 13,292 mg GAE/100 g leaf material). Among *Elliot*, *Duke* and *Spartan*, there were no significant differences for TPC. As observed from the HPLC analysis, the *Toro* and *Nelson* leaf varieties registered with the highest levels for almost all of the individual phenolic compounds. The study of Routray and Orsat [71] investigated the influence of harvest time (May, July, September, and October) on the phytochemical composition of North American highbush blueberry leaves (*Nelson* and *Elliot* varieties). The phenolic content started increasing in September and October, reaching in September 105.204 ± 3.826 mg GAE/g dry matter (*Nelson)* and 120.962 ± 1.420 mg GAE/g dry matter (*Elliot*). However, the highest quantity of phenolic compounds was observed in October, with similar amounts for both varieties (152.356 ± 3.369 mg GAE/g dry matter for *Nelson* and 155.830 ± 2.103 mg GAE/g dry matter for *Elliot*). These results are opposite to our findings, considering that *Nelson* leaves had a higher amount of TPC than *Elliot*, but on the same range considering the amounts. The explanation could be supported by the differences in the geographical areas; precisely, the different habitat of these varieties. When compared to blueberry fruits, the leaves possess 10-fold higher levels of TPC, according to the range of values reported by Li et al. [24] for different blueberries cultivars, namely 154.7 ± 1.01 mg 100 GAE g‒1 FW (*Northland*) and 398.0 ± 5.8 mg GAE 100 g‒1 FW (*Puru*).

The TFCs of the leaf extracts of all the six varieties are presented in Figure 1B. *Toro* leaves registered the highest level, precisely 6788 mg QE/100 g leaf material, followed by the *Spartan* variety, with a 1.5-fold lower level. The *Nelson* variety registered the lowest level of TFC (3136 mg QE/100 g leaf material), whereas *Bluecrop* and *Elliot* leaves presented no significant differences between their TFC. According to the recent study of Li et al. [24], where 13 cultivars of half-bush and highbush blueberries were investigated, the total flavonoid content ranged from 161.7 ± 3.2 mg QE 100 g‒1 FW in *Puru* to 512.3 ± 4.3 mg QE 100 g‒1 FW in *Bluecrop*. Therefore, we can conclude that the leaves of the blueberry are richer in flavonoids than their counterpart fruits.

In many blueberries studies, the TFC was significantly higher when compared to the TPC. A possible explanation may be derived from the TPC assay used, as an overestimation of TPC may arise due to other substances’ interference, such as the existence of the nonphenolic components in the extract [72].

The TPC and TFC results were correlated with the ones of the DPPH assay so that a correlation can be achieved between the antioxidants and the antioxidant capacity. As illustrated in Figure 1A,B,D, the *Toro*, *Elliot*, and *Nelson* varieties registered the highest TPC and TFC values—a fact which is reflected in their highest DPPH activity (D). The positive correlations between the DPPH antioxidant assay and the phenolics content suggest that the phenolic compounds present in ultrasound-assisted ethanolic extractions have a strong contribution to the antioxidant activity.

### 3.3. Total Anthocyanins Content

The total anthocyanin content is displayed in Figure 1C. Following the HPLC analysis, only the *Toro*, *Elliot*, and *Spartan* leaf varieties have shown any anthocyanin content. There were significant differences among the cultivars; *Toro* leaves had the highest TAC (16.81 mg/100 g leaf material), and the Spartan variety the lowest (9.55 mg/100 g leaf material). Wang et al. [40] reported that the TAC of blueberry fruits was ten times higher than that of the leaves, (0.09–4.4 mg cyanidin 3-O-glucoside equivalents/g dry weight). However, some cultivars had no anthocyanin content, which is in line with our findings. In the study of Routray and Orsat [71], the TPC pattern was also similar for the TAC and antioxidant activity for two cultivars (*Nelson* and *Elliot*)—precisely, a higher content in September and October, with *Elliot* coming first. These findings match ours, considering that the *Toro* variety registered the highest TPC, TAC and DPPH activity. In another piece of research [69], the geographical variation of anthocyanins in some blueberries varieties—including *Duke*, *Bluecrop*, and *Spartan*—was investigated, wherein the cultivar and climatic factors were reported to have a significant influence in the proportions of each anthocyanin.

### 3.4. DPPH Radical Scavenging Activity

One of the needed features of an antioxidant is its capacity to counteract radical-induced oxidative stress. The antioxidant molecule should react against the radical and result in antioxidant radicals. To evaluate the radical scavenging of the leaves from the six Romanian blueberry varieties, reactions of DPPH radicals with the extracts were performed. The results of the DPPH antioxidant activity analysis are shown in Figure 1D. The *Toro* and *Elliot* cultivars registered the highest percentage inhibition of DPPH radicals (70.41% and 68.42%, respectively), followed by *Nelson* leaves (58.69%). There were significant differences between the antioxidant capacities among all of the cultivars, with *Duke* leaves as the poorest source of antioxidants. Overall, the cultivars *Toro*, *Elliot*, and *Nelson* appear to be good sources of antioxidants.

In the existing literature, the antioxidant capacity was statistically correlated with TPC, TFC, and TAC, indicating that the polyphenols have a significant role in the antioxidant activity [25,47,73,74]. Therefore, the *Toro*, *Elliot*, and *Nelson* varieties, with their higher polyphenolic content, may deliver a greater range of antioxidants, being valuable cultivars for production, research, and health benefits.

The rich phenolics composition in blueberry leaves was correlated to their antioxidant activities [35]. In line with these results, Ferlemi et al. [68] demonstrated the strong antioxidant potential of the *V. corymbosum* leaf. The study of Wang et al. [67] investigated the polyphenolic composition and antioxidant activities in the leaves of blueberry cultivars. The DPPH, ABTS+, and FRAP results demonstrated that the antioxidant activities of most rabbiteye blueberry cultivars were generally higher than those of highbush varieties.

Numerous plant-derived antioxidant compounds have been used in medical and pharmaceutical products for replacing artificial antioxidants, as the latest research has underlined their potential negative role in carcinogenesis [75].

### 3.5. Evaluation of the Antimicrobial Activity

All of the blueberry leaf varieties have been tested for their antibacterial and antifungal capacity against several strains, selected based on their relevance in the health and food sectors. Table 3 presents the results of MIC, and Table 4 the results of MBC/MFC. Significant antimicrobial effects were registered in all six cultivars’ leaf extracts, depending on the tested strain: Gram-positive, Gram-negative, or fungi.

Regarding the Gram-positive bacteria, the highest sensitivity was registered by *Rhodococcus equi*, whereas all the six cultivars’ leaves had the same—precisely, the highest inhibitory effect (MIC = 0.06 and MBC = 0.12 mg/mL). The *Staphylococcus aureus* and *Enterococcus faecalis* strains were not as sensitive as *Rhodococcus equi.* Towards *Staphylococcus aureus*, *Toro* and *Spartan* varieties registered the highest antibacterial potential (MIC = 0.06 and MBC = 0.12 mg/mL), followed by *Elliot*, *Nelson*, and *Bluecrop* (MIC = 0.12 and MBC = 0.24 mg/mL). The *Enterococcus faecalis* strain was the most resistant to all six cultivars’ extracts, whereas, *Toro*, *Elliot*, *Spartan*, and *Nelson* inhibited the most (MIC = 0.12 and MBC = 0.24) when compared to the control and the other varieties. These findings may be explained by the highest amount in TPC, TFC, and TAC of *Toro* variety, the same TPC level for *Nelson* variety, and the high TFC and TAC values for the *Elliot* and *Spartan* varieties, considering the evidences on polyphenols’ roles in the antimicrobial activity of natural extracts—precisely, certain interactions between polyphenols and bacterial cell surfaces, and the derived antimicrobial effect [76].

In the case of the Gram-negative strains, the highest sensitivity was reported for *Klebsiella pneumonia*, whereas all of the varieties, except *Bluecrop*, had the highest inhibitory capacity (MIC = 0.12 and MBC = 0.24 mg/mL). The *Pseudomonas aeruginosa* strain was the most resistant towards the extracts’ inhibitory effects, whereas the minimum inhibitory concentration registered was 0.24 (mg/mL) for three cultivars—precisely *Elliot*, *Nelson*, and *Bluecrop*—while *Toro* and *Spartan* leaf extracts registered the lowest antibacterial effect (MIC = 1.92 and MBC = 3.84 mg/mL). Towards the Gram-negative bacterium *Escherichia coli enterotoxigen,* all of the six varieties’ extracts had the same inhibition capacity (MIC = 0.48 and MBC = 0.96 mg/mL). Overall, the *Toro* leaves, probably due to their higher phenolic content and higher antioxidant activity, exhibited the highest inhibitory results towards all the strains, except *Pseudomonas aeruginosa*, closely followed by *Elliot* and *Nelson* varieties. Our results also suggest that the Gram-negative bacteria are more resistant to the leaf extracts than Gram-negative bacteria. Based on the literature, the strongly-manifested antimicrobial mechanism of phenolics is considered to rely on the cytoplasmic membrane, and the existence of an extra lipidic membrane for the Gram-negative bacteria may give rise to a stronger protection mechanism [77], and therefore higher resistance.

According to the existing literature, highbush blueberry leaf extracts proved to have a good antimicrobial capacity, mostly towards *Salmonella typhymurium* and *Enterococcus faecalis* [78]. The antimicrobial capacity of the blueberry extracts was reported towards a range of strains. Silva et al. [48] reported that the blueberry leaf extract had antimicrobial effects on *Staphylococcus aureus*, *Salmonella enteritidis*, *Enterococcus faecium*, *Listeria innocua*, and *Bacillus cereus*. The assay implied the determination of the MIC and MBC. In the same study, the antimicrobial capacity of blueberry leaves was compared to those of the fruits, and it was reported that the leaf extracts had the highest activity [48].

Furthermore, the hydroalcoholic blueberry leaf extracts were tested for their antimicrobial capacity [78], whereas the disk diffusion method was applied towards Gram-negative bacteria—precisely, *Escherichia coli*, *Pseudomonas aeruginosa*, *Salmonella typhimurium*, *Acetobacter baumannii*, and *Klebsiella pneumoniae*—and Gram-positive bacteria, such as *Staphylococcus aureus* and *Enterococcus faecalis.* The findings showed an antimicrobial inhibition of all of the tested bacteria by the blueberry leaf extracts. More precisely, a 5 mg/disc of the extract showed an inhibition zone with a diameter ranging between 8.37, for *Escherichia coli*, and 16.67, for *Salmonella typhimurium.* The 10 mg/disc of the extract increased the range of the diameters for the inhibition zone to between 14.08, for *Enterococcus faecalis*, and 23.18 for *Salmonella typhimurium* [78].

Regarding the antifungal capacity, all of the cultivars had no effect against *Candida albicans* (MIC = 125 and MFC = 250 mg/mL), in comparison with the control Fluconazole (MIC = 15.62 and MFC = 31.25 mg/mL), *Candida parapsilosis* (MIC = 31.25 and MFC = 62.5) was the most sensitive. Towards *Candida zeylanoides*, the highest antifungal activity was reported for the *Bluecrop* variety (MIC = 31.25 and MFC = 62.5 mg/mL), whereas all of the other five varieties showed the same antifungal effect (MIC = 62.5 and MFC = 125 mg/mL). Comparing the three fungi, *Candida parapsilosis* was the most sensitive; therefore, it could be stated that the six Romanian varieties have a fungi-dependent antifungal effect.

### 3.6. Evaluation of the Antimutagenic Capacity of Romanian Blueberry Cultivars

The plant’s antimutagenic properties are of major interest in pharmaceutical and medical applications, as the latest research has underlined the key role of mutation in carcinogenesis [79]. The leaf extracts belonging to the six Romanian blueberry varieties were tested for their antimutagenic capacity, starting from the findings reported in our recent review concerning their valuable antioxidant potential [22]. The results are presented in Table 5 below.

Regarding the *Salmonella typhimurium* TA98, the leaf extracts of half of the tested cultivars significantly inhibited the TA98 strain. Moderate inhibition (25–40%) was registered by the *Toro*, *Spartan*, and *Bluecrop* varieties; the *Toro* leaves showed the highest inhibition percentage (32.98%). These findings might be explained by the highest scavenging capacity registered by *Toro*, considering that the literature reported a strong correlation between antioxidant capacity and the antimutagenic activity of plant extracts [80]. Moreover, the *Spartan* variety registered the second-highest TFC, and was among the three varieties (including *Toro*) with TAC values, aspects that might contribute to their antimutagenic effect. According to the method, a value below 25% is considered low/no inhibition; therefore, we can state that *Elliot* (19.07%), *Duke* (21.64%), and *Nelson* (22.16%) had no antimutagenic activity towards TA98.

Concerning TA100, all of the leaf extracts from the six varieties showed a high antimutagenic inhibition, indicating that *Salmonella typhimurium* TA100 was not as resistant to blueberry leaf extracts as TA98. According to Table 5, all of the leaf cultivars registered a moderate inhibitory capacity (25–40%), whereas the highest inhibition percentage was exhibited by *Toro* leaf (38.68%) extract, closely followed by *Duke* (36.67%) and *Bluecrop* (35.81%) extracts. The lowest inhibition percentage (27.22%) was exhibited by the *Nelson* variety.

In the case of both strains, the antimutagenic effects could be linked to a high content of flavanols and flavonols, considering the evidence on flavonoids and decreased mutagenic activity of the standard mutagens. Based on our knowledge, the antimutagenic evaluation of leaves from commercial blueberry cultivars has never been the subject of a research paper; therefore, this has valuable novelty for the current knowledge in the field. Besides this, the literature is poor in studies evaluating this specific features of blueberry fruits or/and leaves. In the study of Smith et al. [26], the antimutagenic capacity of several berries was evaluated, including the rabbiteye blueberry, the Tifblue cultivar, and the Premier cultivar. The reported antimutagenic inhibition was 24% for the Premier cultivar in ethanol anthocyanin-rich extract, and 34% in methanol extract, while for the Tifblue cultivar, it was 43% in ethanol anthocyanin-rich extract. Considering the recent review paper on flavonoids’ bioactivity [81], the plant flavonoids contribute significantly to the antimutagenic capacity.

Based on our findings, the leaf extracts of the six Romanian cultivars do have a significant content of phenolic compounds, with a possible role in the biological profile of the tested hydroethanolic extracts. Moreover, they also contain procyanidins, which are well-known for their good antioxidant capacity, with direct impact in both in vitro and in vivo antibacterial and antimutagenic capacities [22]. These findings underline these leaves’ potential as a safe and useful alternative for the prevention of mutations, and as a promising source of antimutagenic compounds.

The first step in cancer formation is damage occurring to the genome of a somatic cell, resulting in a mutation in an oncogene or a tumor-suppressor gene. Therefore, the ability to inhibit the production of mutations by different direct-acting mutagens is an important step in the use of plant extracts in medical applications, such as cancer prevention [26]. Moreover, as flavonoids can support apoptosis in different cancer cells, in particular, quercetin represents an important anticancer compound against prostate and breast cancers [82,83].

## 4. Conclusions

In summary, the overall results suggest that blueberry leaf extracts are a rich source of potent phenolic antioxidants. Our findings have demonstrated that, whilst they are detrimental to several controlled environmental factors (soil, irrigation) and have only one growing season, cultivars significantly influence the phenolic composition and content, and the antioxidant and antimutagenic capacities of *V. corymbosum* L. leaves, whereas the antimicrobial activity was mostly strain-dependent. However, other stress factors, such as temperature and solar exposition, had their particular influence in the polyphenolic synthesis, but were not investigated in the present paper. The findings regarding the phenolic compounds indicated that even whilst they are similar for all of the six cultivars, the concentrations and proportions differ, whereas anthocyanins were found only in half of the examined varieties leaves. The best-represented subclasses in terms of the number of phenolic compounds were flavanols and flavonols, with rutin as the major compound, while the most abundant group in terms of the highest levels, for all six varieties, was represented by hydroxycinnamic with feruloylquinic acid as the major compound. *Toro*, *Elliot*, and *Nelson* had higher antioxidant capacities than the other three cultivars. Blueberry leaf extracts had high antibacterial activity and less antifungal capacity, and significant antimutagenic capacity towards *Salmonella typhimurium* TA98 and TA100 strains, with *Toro* leaf as the best candidate. This study contributes to the current knowledge in the field of the blueberry leaf’s chemical and biological profile, exploring the most commercialized varieties, offering alternative sources of health-related compounds for the medical and pharmaceutical sectors.

## Figures and Tables

**Table 1:** Identification of the phenolic compounds from the flavonoid class (flavanols, flavonols, and anthocyanins) and hydroxycinnamic acids class in the leaf extracts of the blueberry varieties.

Peak No.	Retention Time R_t_ (min)	UV λ_max_ (nm)	[M + H] ^+^ (m/z)	Compound	Subclass
1	2.97	279	307, 290	Gallocatechin	Flavanol
2	11.33	280	579, 291	Procyanidin dimer I	Flavanol
3	12.01	281, 329	355, 163	5-Caffeoylquinic acid (Chlorogenic acid)	Hydroxycinnamic acid
4	12.58	280	291	Catechin	Flavanol
5	13.11	280	291	Epicatechin	Flavanol
6	13.41	282, 329	181, 163	Caffeic acid	Hydroxycinnamic acid
7	13.89	280	865, 291	Procyanidin trimer	Flavanol
8	14.79	283, 330	369	Feruloylquinic acid I	Hydroxycinnamic acid
9	15.35	263, 355	611, 303	Quercetin-rutinoside (Rutin)	Flavonol
10	16.20	263, 355	465, 303	Quercetin-glucoside	Flavonol
11	17.83	263, 356	493, 303	Quercetin-acetyl-rhamnoside	Flavonol
12	18.69	262, 355	435, 303	Quercetin-arabinoside	Flavonol
13	19.74	280	579, 291	Procyanidin dimer II	Flavanol
14	20.08	282, 329	517, 163	Dicaffeoylquinic acid	Hydroxycinnamic acid
15	21.15	263, 355	628, 303	Quercetin-diglucoside	Flavonol
16	21.88	261, 355	303	Quercetin	Flavonol
17	11.02	210, 517	449, 287	Cyanidin-glucoside	Anthocyanins
18	11.78	214, 517	419, 287	Cyanidin-arabinoside	Anthocyanins
19	14.28	218, 518	491, 287	Cyanidin-acetyl-glucoside	Anthocyanins

**Table 2:** The content of individual phenolic compounds in the leaf extracts of the six blueberry varieties, using HPLC-MS and expressed as mg/g.

Phenolic Compounds		Cultivars					
		*Elliot*	*Toro*	*Duke*	*Bluecrop*	*Spartan*	*Nelson*
**Flavanols**	Gallocatechin	8.52 ± 0.07 ^b^	9.03 ± 0.08 ^a^	4.84 ± 0.05 ^e^	4.90 ± 0.05 ^e^	7.18 ± 0.06 ^c^	6.73 ± 0.08 ^d^
	Catechin	6.46 ± 0.07 ^b^	7.97 ± 0.07 ^a^	6.03 ± 0.07 ^c^	5.21 ± 0.05 ^d^	6.15 ± 0.05 ^c^	4.87 ± 0.08 ^e^
	Epicatechin	4.19 ± 0.03	n.d	n.d	n.d	n.d	n.d
	Procyanidin dimer I	3.69 ± 0.04	n.d	n.d	n.d	n.d	n.d
	Procyanidin dimer II	12.50 ± 0.1 ^c^	14.13 ± 0.1 ^b^	12.30 ± 0.1 ^d^	12.41 ± 0.1 ^cd^	17.15 ± 0.1 ^a^	3.92 ± 0.03 ^e^
	Procyanidin trimer	6.36 ± 0.08 ^f^	15.99 ± 0.2 ^a^	7.02 ± 0.12 ^d^	8.98 ± 0.10 ^c^	6.80 ± 0.09 ^de^	12.27 ± 0.1 ^b^
**Hydroxycinnamic acids**	Chlorogenic acid	0.90 ± 0.01 ^c^	1.23 ± 0.02 ^a^	0.52 ± 0.01 ^d^	0.54 ± 0.01 ^d^	0.44 ± 0.01 ^e^	1.03 ± 0.01 ^b^
	Caffeic acid	5.93 ± 0.06 ^a^	4.75 ± 0.03 ^c^	4.49 ± 0.05 ^d^	2.62 ± 0.02 ^f^	3.61 ± 0.02 ^e^	5.36 ± 0.05 ^b^
	Feruloylquinic acid	40.94 ± 0.3 ^c^	44.43 ± 0.3 ^b^	29.15 ± 0.2 ^d^	25.36 ± 0.2 ^e^	19.23 ± 0.1 ^f^	49.62 ± 0.4 ^a^
	Dicaffeoylquinic acid	7.99 ± 0.07 ^b^	7.30 ± 0.09 ^c^	4.23 ± 0.05 ^d^	2.73 ± 0.02 ^e^	4.58 ± 0.03 ^d^	10.18 ± 0.1 ^a^
**Flavonols (quercetin derivatives)**	Quercetin-rutinoside (Rutin)	31.53 ± 0.1 ^c^	35.77 ± 0.1 ^a^	17.64 ± 0.1 ^e^	19.10 ± 0.1 ^d^	14.44 ± 0.1 ^f^	32.50 ± 0.2 ^b^
	Quercetin-glucoside	3.46 ± 0.04 ^b^	6.70 ± 0.08 ^a^	2.86 ± 0.02 ^d^	3.54 ± 0.03 ^b^	3.08 ± 0.03 ^c^	2.09 ± 0.02 ^e^
	Quercetin-acetyl-rhamnoside	1.22 ± 0.01 ^b^	1.92 ± 0.02 ^a^	0.45 ± 0.01 ^e^	0.68 ± 0.01 ^d^	0.46 ± 0.01 ^e^	0.78 ± 0.01 ^c^
	Quercetin-arabinoside	n.d	n.d	n.d	n.d	1.40 ± 0.01	n.d
	Quercetin-diglucoside	0.35 ± 0.01 ^b^	0.53 ± 0.01 ^a^	n.d	n.d	n.d	n.d
	Quercetin	2.70 ± 0.02 ^c^	3.68 ± 0.04 ^a^	2.25 ± 0.06 ^d^	2.07 ± 0.04 ^e^	3.69 ± 0.02 ^a^	3.56 ± 0.02 ^ab^
**Anthocyanins**	Cyanidin-glucoside	0.63 ± 0.01 ^b^	1.08 ± 0.08 ^a^	n.d	n.d	0.52 ± 0.01 ^b^	n.d
	Cyanidin-arabinoside	0.41 ± 0.01 ^b^	0.80 ± 0.01 ^a^	n.d	n.d	0.42 ± 0.01 ^b^	n.d
	Cyanidin-acetyl-glucoside	0.32 ± 0.01 ^a^	0.35 ± 0.01 ^a^	n.d	n.d	0.26 ± 0.01 ^b^	n.d
**Total phenols**		138.09 ^b^	155.67 ^a^	92.46 ^d^	88.14 ^e^	89.40 ^e^	132.91 ^c^

In the same rows, the values (means ± SD, mg/g, *n* = 3) marked by different letters (a–f) report significant differences (*p* < 0.05) among the six varieties (One-way ANOVA-Tukey multiple range test (*p* = 0.05)). n.d—not detected.

**Table 3:** Minimum inhibitory concentration (MIC) of leaf extracts from the six Romanian blueberry varieties, expressed as mg/mL.

	Type of Strains	Gram-Positive			Gram-Negative			Fungi		
**Sample**	**Varieties**	*S. aureus*	*E.* *fecalis*	*R. equi*	*E. coli enterotoxigen*	*K. pneumonia*	*P. aeruginosa*	*Candida* *albicans*	*Candida* *zeylanoides*	*Candida* *parapsilosis*
	mg/mL									
**Leaves**	*Elliot*	0.12	0.12	0.06	0.48	0.12	0.48	125	62.5	31.25
	*Toro*	0.06	0.12	0.06	0.48	0.12	1.92	125	62.5	31.25
	*Duke*	0.24	0.24	0.06	0.48	0.12	0.96	125	62.5	31.25
	*Bluecrop*	0.12	0.24	0.06	0.48	0.24	0.48	125	31.25	31.25
	*Spartan*	0.06	0.12	0.06	0.48	0.12	1.92	125	62.5	31.25
	*Nelson*	0.12	0.12	0.06	0.48	0.12	0.48	125	62.5	31.25
**Control**	Fluconazole μg/mL	-	-	-	-	-	-	15.62	7.81	15.62
	Streptomicyn μg/mL	0.03	0.06	0.06	0.12	0.06	0.06	-	-	-

**Table 4:** Minimum bactericidal concentration (MBC/MFC) of the leaf extracts from the six Romanian blueberry varieties, expressed as mg/mL.

	Type of Strains	Gram-Positive			Gram-Negative			Fungi		
**Sample**	**Varieties**	*S. aureus*	*E.* *fecalis*	*R. equi*	*E. coli enterotoxigen*	*K. pneumonia*	*P. aeruginosa*	*Candida* *albicans*	*Candida* *zeylanoides*	*Candida* *parapsilosis*
	mg/mL									
**Leaves**	*Elliot*	0.24	0.24	0.12	0.96	0.24	0.96	250	125	62.5
	*Toro*	0.12	0.24	0.12	0.96	0.24	3.84	250	125	62.5
	*Duke*	0.48	0.48	0.12	0.96	0.24	1.92	250	125	62.5
	*Bluecrop*	0.24	0.48	0.12	0.96	0.48	0.96	250	62.5	62.5
	*Spartan*	0.12	0.24	0.12	0.96	0.24	3.84	250	125	62.5
	*Nelson*	0.24	0.24	0.12	0.96	0.24	0.96	250	125	62.5
**Control**	Fluconazole μg/mL	-	-	-	-	-	-	31.24	15.62	31.24
	Streptomicyn μg/mL	0.06	0.12	0.12	0.24	0.12	0.12	-	-	-

**Table 5:** Antimutagenic activity.

Sample	Varieties	Number of Revertants			
		TA98		TA100	
		Mean ± S.D	Inhibition %	Mean ± S.D	Inhibition %
**Leaves**	Negative control	9.35 ± 3.2 ^a^		9.35 ± 2.1 ^a^	
	*Elliot*	157 ± 5.4	19.07	250 ± 6.5	28.36
	*Toro*	130 ± 5.9	32.98	214 ± 3.2	38.68
	*Duke*	152 ± 7.8	21.64	221 ± 6.5	36.67
	*Bluecrop*	142 ± 8.6	26.8	224 ± 4.4	35.81
	*Spartan*	139 ± 5.7	28.35	232 ± 4.1	33.52
	*Nelson*	151 ± 6.2	22.16	254 ± 4.4	27.22
	4-NPD ^b^	194 ± 3.3	-	-	-
	NaN_3_^b^	-	-	349 ± 15.22	-

^a^ Values expressed are means ± SD of three replications. ^b^ 4-NPD and NaN_3_ were used as positive controls for *Salmonella thyphimurium* TA98 and TA100 strains, respectively.
